# Impact of patient information leaflets on pain medication intake behavior: a pilot study

**DOI:** 10.1097/PR9.0000000000000620

**Published:** 2017-09-29

**Authors:** Julia Schmitz, Sandra Kamping, Janine Wiegratz, Maike Müller, Jan Stork, Luana Colloca, Herta Flor, Regine Klinger

**Affiliations:** a Department of Anesthesiology, Section Pain Medicine and Pain Psychology, University Medical Center Hamburg-Eppendorf, Hamburg, Germany; b Department of Anesthesiology, University Hospital of Würzburg, Würzburg, Germany; c University of Maryland School of Nursing, University of Maryland School of Medicine, Baltimore, MD, USA; d Department of Cognitive and Clinical Neuroscience, Central Institute Mental Health, Medical Faculty Mannheim, Heidelberg University, Mannheim, Germany

**Keywords:** Clinical application, Placebo, Nocebo, Expectancy, Patient information leaflet

## Abstract

**Introduction:**

Patient information leaflets on pain medication primarily list side effects while positive effects and action mechanisms remain underrepresented. Nocebo research has shown that negative instructions can lower analgesic effects.

**Objectives:**

Research on information leaflets and their influence on mood, memory of side effects, and intake behavior of healthy participants is needed.

**Methods:**

To determine the ratio of positive to negative phrases, 18 information leaflets of common, over-the-market analgesics were examined of which 1 was selected. In a randomized, controlled study design, 18 healthy participants read this leaflet while 18 control group participants read a matched, neutral leaflet of an electrical device. Collected data concerned the recall of positive and negative contents, mood, anxiety, and the willingness to buy and take the drug.

**Results:**

All examined leaflets listed significantly more side effects than positive effects (t_17_ = 5.82, *P* < 0.01). After reading the analgesic leaflet, participants showed a trend towards more negative mood (*F*_1,34_ = 3.78, *P* = 0.06, η_p_^2^ = 0.1), a lower intention to buy [χ^2^ (1, n = 36) = 12.5, *P* < 0.01], a higher unwillingness to take the medication [χ^2^ (1, n = 36) = 7.2, *P* < 0.01], and even a greater recall for side effects than positive effects (t_17_ = 7.47, *P* < 0.01).

**Conclusion:**

Reading the patient information leaflets can increase fear and lower the intention to buy and the willingness to take a pain medication.

## Abstract 2

Negative wording in package information leaflets of pain medication is associated with a tendency to evoke negative emotions and lower willing to take medication.

## 1. Introduction

Package leaflets of pain medication commonly emphasize the negative (eg, side effects) instead of the positive effects (eg, analgesic modes of action and the expected reduction of pain). In Germany, every prescribed or over-the-counter analgesic has to come with a package leaflet. Little is known about how patient information leaflets (PILs) may affect mood and the intention to use and take the drug. Evidence from placebo and nocebo research indicates that the information on the medication influences expectations and ultimately changes the reaction to the drug.^7,9,23,25,29^ Bingel et al.^11^ showed that the effectiveness of the pain medication decreased once the patients were told that their pain relief was expected to be low. Other studies have revealed that negative and anxiety-inducing information can foster the occurrence of side effects^18,28,39^ and, in the case of analgesics, can increase pain.^11^

One underlying mechanism for this negative influence of information (nocebo effect) could be a selective attention towards new and personally unpleasant sensations^36^ that may be falsely attributed to the medication.^6^ Colloca et al. showed that a nonpainful tactile stimulation was considered painful when introduced by negative verbal anticipations of pain.^21^ Therefore, negative verbal instructions can provoke a nocebo effect. Conversely, positive instructions can lead to greater pain tolerance. When participants were told that treating their hand with ice water would raise their blood circulation and cell renewal levels, they could keep their hand in ice water longer than the control group (CG).^48^

Negative verbal suggestion may also lead to anxiety or fear. While Colloca and Benedetti have described the neurobiological mechanism of how anxiety can turn into pain,^17^ Flaten et al.^27,40^ concentrated on subjective ratings of pain, showing that fear and negative emotions can increase pain perception. According to the commonly cited fear-avoidance model of pain,^3,22,38,51^ a person who (negatively) believes that physical movement increases pain will ultimately move less and experience more pain. Consequently, when patients are afraid of side effects, they can either avoid taking the drug or develop a negative attitude towards the drug and expect a negative outcome. This negative belief can decrease the drug's effectiveness.

Our study aimed at investigating (1) how many positive or negative effects are presented in the package leaflets of commercially available analgesics; (2) how a package information leaflet (which contains more side effects than positive effects) influences mood, memory of side effects, and intake behavior of healthy participants occasionally suffering from pain. We compared the experimental group (PIL) with a CG that read a neutral leaflet of an electrical device. We expected the readers of the PIL to experience an increase in fear and a decrease in mood and an unwillingness to use the described treatment compared to the CG reading the neutral leaflet.

## 2. Methods

All patients gave their written informed consent. The study was conducted in accordance with the Declaration of Helsinki. It was part of a research grant (DFG RK Kl 1350/3-1). The study consisted of 2 phases: part 1 analyzing the package leaflets and part 2 testing the impact of PILs on medication intake behaviors.

### 2.1. Part 1: analysis of the package leaflets

In this part of the study, the package leaflets of 18 analgesics (6 opioids, and 12 non-opioids) were examined. The basis for selecting the analgesics was the globally established 3-Step plan of the WHO (Word Health Organization)^1^ in treatment of chronic pain. The WHO recommends nonopioid to strong opioids medication. We compared this recommendation with the national S3-guidelines of the German chapter of the International Association for the Study of Pain (IASP; German Chapter: Deutsche Schmerzgesellschaft, German Pain Association) and the Association of the Scientific Medical Societies (AWMF in Germany) “Long-term use of opioids for the treatment of non-tumor-related pain” (LONTS).^4^ Subsequently, we compared these recommendations of the WHO and LONTS with the drug report of prescribed and taken medications of a leading health insurance that insures more than 8.3 Million people (in Germany).^5^ We chose those analgesics that overlapped the most.

The words on the package inserts were counted with regard to their positive connotations (effects of the medication, mode of action of the medication, and positive information on the therapeutic effect of the medication) and negative connotations (side effects). Examples of positive phrasings were “clinical efficacy and the entailed pain relief is highly important,” “clinical studies have verified that this medication effectively reduces pain,” “it will help you to relieve your pain,” and a negative phrasing was, for instance “nausea” or “obstipation.”

#### 2.1.1. Statistical analysis

For part 1, we compared the proportion of negative and positive side effects using independent *t* test.

### 2.2. Method

#### 2.2.1. Part 1: selection of the package leaflets

In the first part of this study, the positive effects and the side effects of the individual package information leaflets were counted and compared. We compared positive and negative information provided in the leaflets of Diclofenac, a nonsteroidal anti-inflammatory drug taken or applied to reduce inflammation and as an analgesic reducing pain in certain conditions.

**Figure 1.**
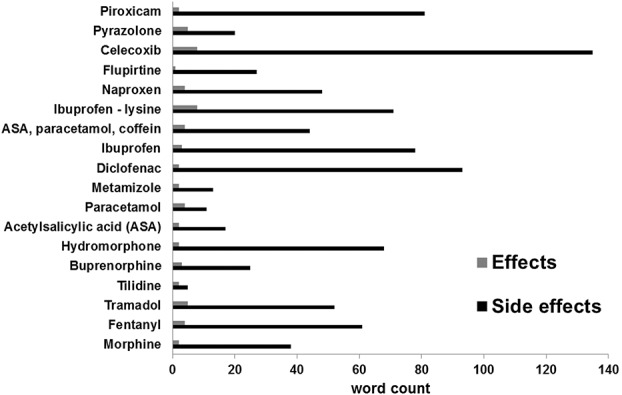
Number of words depicting side effects or effects of 18 package information leaflets of common analgesics.

The distribution of negative and positive word content taken from the respective package leaflets is presented in Figure 1.

An overall ratio of positive to negative phrasings was then attained for all 18 package inserts. A word count of those describing positive and negative effects in total showed a mean of M = 52.78 (SD = 18.67). Based on this number the side effects averaged out at M = 49.28 (SD = 34.24; 93.3%) and the effects at M = 3.5 (SD = 2.01; 6.7%). There was a significant difference between the number of negative (side effects) compared to positive effects (t_17_ = 5.82, *P* < 0.01) with a ratio of 5:1 of negative vs positive aspects. Diclophenac was the prescription drug with the highest rate of negative phrasings and was therefore selected for the second part of the study.

### 2.3. Part 2: impact of patient information leaflets on medication intake behavior

#### 2.3.1. Study population

The study was conducted at the University of Hamburg, in the facilities of the Outpatient Clinic for Behavior Therapy, Department of Psychology. Thirty-six healthy subjects participated in the second part of the study. Eighteen were randomly assigned by drawing lots to the experimental group (PIL group), and 18 participants were assigned to the CG. The groups matched for sex (*F*_1,35_ = 1.06, *P* = 0.31). The groups did not differ significantly in age (*F*_1,35_ = 3.62, *P* = 0.07) and the sample comprised 36.1% male subjects. Participants with chronic diseases were excluded from the study. To be included, participants had to have taken pain medication at least once and had to have suffered from back pain at least once in their lifetime. The 2 groups were not significantly different in frequency of medication intake [χ^2^ (2, n = 36) = 2.61, *P* = 0.27], or whether or not they usually read the leaflet [χ^2^ (1, n = 36) = 1.18, *P* = 0.28]. Demographic data are listed in Table 1.

**Table 1:**
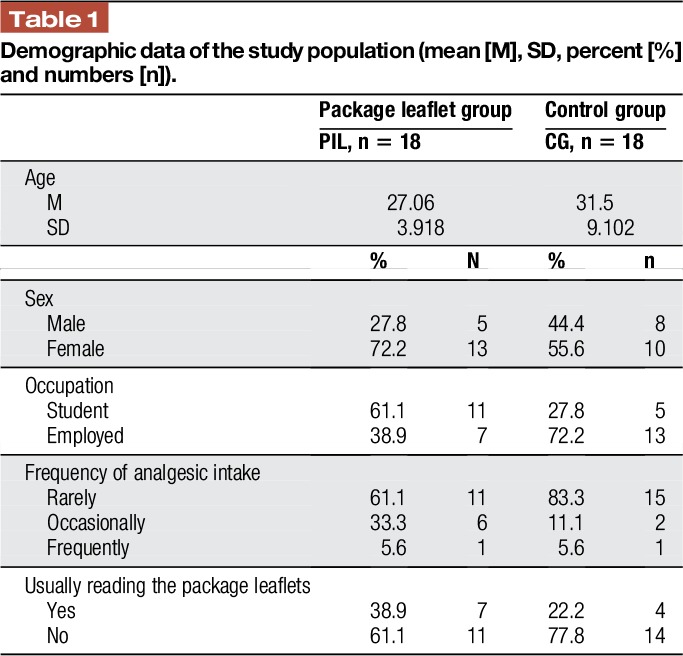
Demographic data of the study population (mean [M], SD, percent [%] and numbers [n]).

#### 2.3.2. Study design

The participants were told that they would take part in a clinical study, which investigated their ability to imagine a situation in which they were experiencing pain. They were informed about the study in accordance with ethical guidelines. First, the participants completed a questionnaire on their mood, general anxiety levels, and their purchase and intake behavior of pain medication (eg, the frequency with which analgesics were taken and the package leaflet read). All participants were then asked to image that they were suffering from severe back pain and were going to a pharmacy to buy the compound diclofenac that was recommended to them by the pharmacist. The experimental group was instructed to read the package leaflet. The CG received a user manual of a vacuum cleaner, of which the first 7 pages were used to match the manual with the length of the package leaflet. The small font size of the package insert was also applied to the vacuum manual. All participants were instructed to carefully read the text as they would be asked questions on the content afterwards. Subsequently, they were asked to imagine that they were standing in the pharmacy again in which they would be receiving and taking the medication. Questions on their willingness to buy and take the medication were asked after reading the respective informational content. The participants of the experimental group were instructed to take 5 minutes to write down any effects or side effects that they remembered from the text. They were also asked how well they were able to imagine the situation and the back pain on a scale from 0 (could not imagine it at all) to 10 (could imagine it very well).

#### 2.3.3. Psychological measurements

To assess mood, we chose the valence scale of the Self Assessment Manikin (SAM),^12^ as it has good statistical values (affective ratings were very reliable (valence, *r* = 0.99; arousal, *r* = 0.93).^37^ Retest reliabilities were highly similar for both affective dimensions (valence, *r* = 0.99; arousal, *r* = 0.97^30^). Previous experiments have shown that this method provides a simple and efficient way to evaluate emotions.^31,42^ Self Assessment Manikin uses pictorial descriptions of positive or negative valence of emotional materials. SAM is used to assess current mood and is transformed to a scale from 1 (bad mood) to 9 (very good mood). Anxiety levels were assessed on a numerical rating scale with the endpoints “no fear to strong fear,” which were transformed into a scale from 0 to 10. Willingness to buy and take the drug was assessed on a 2-tiered scale (yes/no). Participants who had read the package leaflets were asked to spontaneously write down every positive effect and side effect that they could remember. The participants were given 5 minutes for this task. The abilities to imagine the described back pain, and the overall situation were assessed by manipulation checks each ranging on a scale from 0 (weak imagination) to 10 (strong imagination).

#### 2.3.4. Statistical analysis

For part 2, descriptive statistics were attained for sample size, frequencies, mean values, and standard deviations (SDs). The analysis of the dependent variables (mood and anxiety) was conducted using the generalized linear modeling with repeated measure analyses of variance (ANOVAs) containing the within-subject factor “time” (pre-post reading PIL) and the between-subject factor (experimental and control group). For the main hypothesis, we used the χ^2^ test, *t* test and the single-factor ANOVA. Parametric statistics are fairly robust to minor violations of normality. The data were analyzed using IBM SPSS 22.0 and *P* < 0.05 was set as significance level.

## 3. Results

### 3.1. Part 2: primary outcome measures

#### 3.1.1. Mood ratings

At the beginning of the experiment, mood levels did not differ significantly between both groups (PIL: M = 6.28; SD = 1.80; CG: M = 5.61; SD = 1.51; *F*_1,34_ = 1.42, *P* = 0.24). After intervention repeated measurement ANOVA revealed a borderline significant effect of the factors GROUP × TIME (PIL: M = 5.11; SD = 1.2; CG: M = 5.44; SD = 1.5; *F*_1,34_ = 3.78, *P* = 0.06, η_p_^2^ = 0.1).

#### 3.1.2. Anxiety ratings

Prior to the intervention, anxiety levels did not differ significantly between both groups (PIL: M = 1.61; SD = 1.50; CG: M = 1.72; SD = 2.11; *F*_1,34_ = 0.03, *P* = 0.86). After the intervention, repeated measurement ANOVA revealed a marginal not significant effect of the factors GROUP × TIME (PIL: M = 2.78; SD = 2.2; CG: M = 1.83; SD = 1.9; *F*_1,34_ = 3.09, *P* = 0.09, η_p_^2^ = 0.08).

#### 3.1.3. Willingness to buy and take the analgesics

In the PIL group, 61.1% of the participants stated that they do not want to buy the drug or request another medication. In the CG, 5.6% did not want to buy the drug, or requested another. 38.9% of the PIL group wanted to buy the drug, in contrast to 94.4% in the CG. This group difference was significant [χ^2^ (1, n = 36) = 12.5, *P* < 0.01].

The variable “intake behavior” also differed between groups. Following the intervention, 77.8% of the PIL group and 33.4% of the CG would not take the medication. Merely 22.2% of the PIL group stated that they would take the medication, whereas 66.7% of the CG said that they would take it without further concerns [χ^2^ (1, n = 36) = 7.2, *P* < 0.01].

#### 3.1.4. Memory task in the patient information leaflets group

After reading the leaflet, participants were asked to remember all the effects and side effects mentioned in the leaflet. The participants recalled in total 9.45 words (out of 31 words in the leaflet). They remembered significantly more side effects (82.1%) than positive effects (17.9%) of the drug. On average, the participants listed 1.56 (SD = 1.15) positive effects of the drugs (out of a total of 8 mentioned in the leaflet, eg, 19.50%) and 7.89 (SD = 4.48) side effects (out of 23 mentioned, eg, 34.30%) [t (17) = 7.47, *P* < 0.01], see Table 2.

**Table 2:**
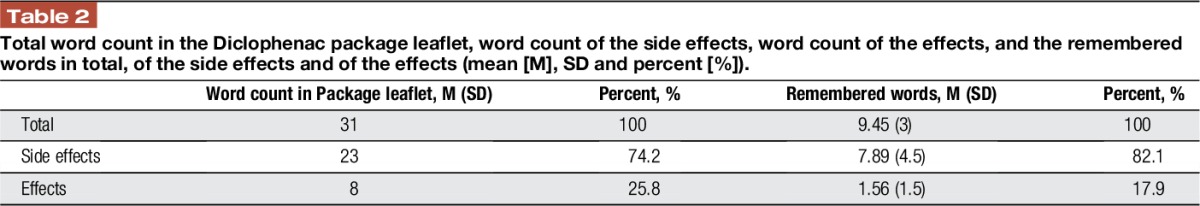
Total word count in the Diclophenac package leaflet, word count of the side effects, word count of the effects, and the remembered words in total, of the side effects and of the effects (mean [M], SD and percent [%]).

#### 3.1.5. Manipulation check

The PIL group exhibited no difficulties in imagining having back pain (M = 6.06; SD = 2.20) and imagining the overall situation (M = 6.28, SD = 2.59). The same was true for the CG (M = 6.50; SD = 2.09 and M = 7.33, SD = 2.45). Both groups did not differ significantly in their capacities in imagining back pain (*F*_1,35_ = 0.38, *P* = 0.54) and the overall situation (*F*_1,35_ = 1.5, *P* = 0.23).

## 4. Discussion

This pilot study with healthy participants examined the impact of reading a PIL after participants imagined being in pain and taking an analgesic drug compared to a CG that imagined reading a neutral user manual. We found that package leaflets contained far more negative than positive informational content. Our results showed a tendency towards mood deterioration and an increase in anxiety levels in the group reading such a PIL, albeit slightly missing significance levels. However, after reading the PIL, the participants did state a decreased willingness to buy and to take the described pain medication.

In what way negative information influences medical effects, especially in analgesics, is of utmost interest. Negative expectancies of an analgesic can reduce its efficacy (nocebo effect).^9,36,45^ Patient information leaflets are an important medium to communicate and influence information concerning analgesics, thereby shaping the expectancies and ultimately the effectiveness of the treatment. Bingel et al.^11^ showed that expectancies that were shaped via positive or negative instructions either increased or decreased the effectiveness of remifentanil accordingly. According to Colloca et al,^18^ every analgesic comprises both a pharmacological and a psychological (placebo) component. This placebo component can be influenced by using positive instructions to induce positive expectations or negative and no instructions to induce negative expectations. The latter could decrease medication effectiveness.^8–10^

Psychological theories that can explain how expectancies transfer into pain are lacking. Turner^50^ proposed anxiety as 1 linking mechanism. The reduction of anticipatory anxiety has also been discussed as a component or cause of the placebo effectiveness.^52^ Expecting pain relief can ameliorate symptoms, because patients may consider the pain problem more controllable. They also may be more likely to notice small improvements, to disregard negative events, and to interpret ambiguous stimuli favorably,^44^ resulting in beneficial behavioral changes, eg, stopping avoidance behavior because of fear of pain. Negative drug information has been shown to increase anxiety and pain intensity levels.^19,24,33,53^ This algesic nocebo led patients to notice even small negative changes in pain, to disregard positive events, and to interpret ambiguous stimuli negatively, resulting in obstructive behavioral changes, eg, anxiety and avoidance behavior by refusing to take the medication. According to our results, only 20% who read the package insert would take the drug. Overrepresenting the side effects in PIL fosters negative emotions (eg, anxious mood after reading the leaflet) resulting in a reduction of the analgesic effect of the drug by causing participants to selectively attend to the negative effects.^16^ Fittingly, Colloca and Benedetti^17^ have shown that when subjects hold the negative belief that their pain will increase, anticipatory anxiety about the forthcoming pain increases, as well. This triggers the activation of cholecystokinin that, in turn, facilitates pain transmission.^17^

For a complete picture, it is of note that the evidence for the effectiveness of nonsteroidal anti-inflammatory drugs is low to very small in chronic back pain.^26^ In low back pain, general systemic medications like opioids and benzodiazepines only have a small and short-term effect on pain.^15^ Especially with regard to the intake of opioids, more than a third of patients is unable to tolerate the side effects and >80% of patients experience adverse events.^14,41^ In this context, it is perhaps unsurprising, given limited data of efficacy (particularly for opioids) and the considerable evidence for intolerable side effects, that information leaflets contain more negative to positive information.

Against this backdrop of both pharmacologically and psychologically caused adverse effects, it is even more important to communicate the effects of the medication in a positive patient–clinician setting^35^ to tailor the information individually and not to leave patients alone with their PILs.^13^ There are 2 basic models to guide health care providers in addressing medication intake. One is the shared decision-making model, in which a paradigm shifts away from the paternalistic patient-clinican relationship, leading to a more patient-centered care in which the patient is more empowered, informed, and autonomous. Research suggests that shared decision-making does influence the way patients consider treatment decisions and may have an impact on outcomes.^46,49^ Another model is the Medication Adherence Model that assumes that 2 types of nonadherence contribute to inconsistent medication intake: the intentional decision to neglect medications and the unintentional interruptions that cause medications not to be taken.^34^ These models are important components in the treatment of patients, but they lack the aspect of self-medication.

The great importance of the PIL in clinical practice is also illustrated by a study conducted by a German statutory health insurance company examining 100 package leaflets of the most commonly prescribed medications.^43^ The survey revealed that 65% of the participants rated the leaflets as the most important source of information regarding effects and side effects of a medication. At the same time, however, 1 of 3 consumers felt unsettled after reading the PIL and nearly a third of the respondents reported having stopped taking the drug or had not even begun taking the drug because of the PIL. Additionally, 42% of the consumers considered the PIL too long and 20% said it was incomprehensible. In addition, a quarter of the foreign terms had not been translated.

There are numerous findings showing that open administration of pain medication (and nonpain medication)^20^ enhances the efficacy of treatments.^2,47^ Clinical implications have already been defined, providing advice on how to enhance placebo and avoid nocebo effects.^36^ For instance, an integral part of open medication is to inform the patient thoroughly: educating the patient on the drugs' action, relativizing negative information, and highlighting positive information.^36^ Putting the side effects into perspective can be used to further explore any preexisting experiences with drug treatments and could additionally lead to an instructed reevaluation of the negative experiences, thereby reducing the nocebo effect (exploration of analgesic-associated experiences and attitudes and reinforcement of positive and devaluation of negative experiences).^36^ Interestingly, educating patients on the nocebo phenomena can be highly beneficial to treatment. A systematic review (4 studies, 400 participants) showed that educating patients with chronic pain by a pharmacist reduced the occurrence of unwanted effects.^32^

The present study and previous insights from placebo and nocebo research should be used to modify the package information leaflets for pain medication. However, the most recent development has been moving in the opposite direction. In 2013, the Federal Institute for Drugs and Medical Devices in Germany introduced a warning triangle on package inserts for drugs whose side effects have not been sufficiently studied. In addition to this warning sign, the leaflets contain even more fear-inducing and negative informational content than before. Patients are explicitly encouraged to report any side effects not described in the package inserts to their doctor, pharmacist, or to the drug administration listed in the package leaflets. Although careful and truthful delineations of side effects are crucial, such a description should be embedded into a context that emphasizes positive drug actions.

Some limitations need to be considered when interpreting the results of the present study. It is based on a small sample of healthy participants without a behavioral correlate. Instead, hypothetical scenarios were used and it seems likely that both the use of intention rather than behavior and the use of hypothetical rather than actual scenarios reduce the validity of test results. Furthermore, the control pamphlet did not contain any emotionally relevant information; a different pamphlet (eg, containing more positive contents) might have provoked more conclusive data points.

Taken together, the results of the study suggest that package information leaflets of commonly used analgesics describe more negative than positive effects of the medication. There also was a trend showing that mood and anxiety levels were negatively influenced by reading these commercially available package leaflets. Most significantly, negative emotions resulted in patients being less willing to buy and take the drug. Clinical implications of this research should be to avoid 1-sided negative information via leaflets of analgesic medication, to explain the mechanisms of action without overemphasizing the side effects, and to balance out negative with positive details while complying with the legal and ethical obligation to inform the patient trustfully.^35^ Further research is required to clarify how package information leaflets can be more patient-friendly to reduce anxiety and negative expectations and to enhance the positive expectations of the medication that they are holding in their hands.

## Disclosures

The authors have no conflict of interest to declare.
